# Usefulness of Delayed Films of Contrast Enema for Detecting Hirschsprung’s Disease

**DOI:** 10.7759/cureus.6339

**Published:** 2019-12-10

**Authors:** Nida Sajjad, Kiran Hilal, Kumail Khandwala, Muhammad Arshad, Nasir Uddin

**Affiliations:** 1 Radiology, Aga Khan University Hospital, Karachi, PAK; 2 Pediatric Surgery, Aga Khan University Hospital, Karachi, PAK; 3 Pathology and Laboratory Medicine, Aga Khan University Hospital, Karachi, PAK

**Keywords:** hirschsprung’s disease, contrast enema, pediatric, delayed film

## Abstract

Background

Contrast enema (CE) in Hirschsprung’s disease (HD) provides a road map to surgeons by ascertaining the transition zone (TZ) and helps in pre-surgical planning. In our institute, we use CE as the initial investigation for HD and carry on till the whole colon is fully distended, followed by a 24-hour abdominal film which is also a part of the international protocol. The main aim of this study was to evaluate the usefulness of this 24-hour delayed film in detecting HD, compare it with gold-standard biopsy results, and to evaluate other imaging features of contrast enema for diagnosis of HD in our tertiary-care hospital in Pakistan.

Methods

This retrospective study was conducted at the Department of Radiology, Aga Khan University Hospital, Karachi. Records of pediatric patients referred for radiological evaluation of symptoms and signs suspicious of HD during the years 2007-2017 were reviewed. A delayed film was labeled positive if the contrast was not completely evacuated when the residual contrast was present till transverse colon and not beyond. Specificity and sensitivity along with positive and negative predictive values were calculated for each finding according to rectal biopsy, taken as the gold standard.

Results

In all, 82 patients met the inclusion criteria out of 111 cases, as they had both biopsy results and delayed 24-hour films. HD was confirmed using rectal biopsy in 56 (43 patients were males and 13 were females) of 82 cases. The most sensitive radiological finding was the transition zone with a sensitivity of 91.07%. The rectosigmoid index was the second most common finding on contrast enema with a sensitivity and specificity of 91.07% and 83.93%, respectively. In all, 59% patients had a positive delayed 24-hour film and were confirmed with having HD on biopsy. The sensitivity, specificity, and positive predictive value of delay in contrast evacuation after 24 hours in our study was 81.25%, 90.91%, and 97.50% respectively.

Conclusion

Contrast enema examinations along with the 24-hour delayed film with mid transverse colon cut-off are optimal for initially investigating HD in a developing nation, and our results show that it correlates well with biopsy. However, rectal biopsy still remains the gold standard for diagnosis.

## Introduction

Hirschsprung’s disease (HD) was first described in 1888 by a Danish pediatrician as severe chronic constipation that resulted in congenital megacolon [1]. It is a result of the failure of the cephalocaudal migration of ganglion cells through the neural crest causing a lack of ganglion cells in the colon. The prevalence of this disease is described to be about one in 5,000 live births with a male-to-female ratio of 4:1 [2]. 

Three investigations are used for the evaluation of patients with the suspicion of HD. These are contrast enema (CE), anorectal manometry and rectal biopsy. Among these, the gold standard is rectal biopsy. Complications associated with colorectal biopsy include bleeding, scar and stricture formation, and perforation along with anesthesia-related risks. In our country, physicians seek non-invasive techniques, such as CE screening which is less invasive, readily available, and provides a prompt diagnosis. CE provides a road map to surgeons by ascertaining the transition zone (TZ) and helps in pre-surgical planning [3-4]. Anorectal manometry is unavailable in most centers in Pakistan. In our institute, we use CE as the initial investigation for HD and carry on till the whole colon is fully distended, followed by a delayed 24-hour abdominal film which is also a part of the international protocol [5-6].

In approximately 90% of HD cases, the transition point is located in the rectosigmoid colon; this condition is known as “short-segment aganglionosis”. On the other hand, in “long-segment aganglionosis” the affected region extends from a more proximal segment of the bowel up to the anorectal junction. The question arises if the transition point of HD is identified in the rectosigmoid region on the enema examination, and whether we really need 24-hour delayed film for further confirmation of our initial findings as this additional imaging increases the radiation exposure to pediatric patients.

Therefore, the main aim of this study was to evaluate the usefulness of a 24-hour delayed film in the detection of HD and other imaging features of contrast enema for diagnosis in our tertiary-care center in Pakistan. 

## Materials and methods

This retrospective study was conducted at the Department of Radiology, Aga Khan University Hospital, Karachi after approval from the Ethical Review Committee. Records of pediatric patients (less than 18 years of age) referred for radiological evaluation of symptoms and signs suspicious of HD at the Aga Khan Hospital, Karachi, Pakistan during the years 2007-2017 were reviewed. 

Patients were included in the study when all radiographs were available (including the 24-hour delayed film), and HD had either been confirmed or excluded by rectal biopsy. The inclusion criteria were delayed passage of meconium beyond 48 hours in neonates with other symptoms like abdominal distension, and in older children with chronic refractory constipation despite medical treatment. Patients who had no 24-hour delayed film available after their CE examination, those with prior surgical history or previous enema examinations, and those with suspected pathologies other than HD were excluded from our study.

The data was extracted from the hospital computerized record system. Demographic data including age and gender were documented from the medical record. A pediatric radiologist, working with two radiology residency program trainees, blindly reviewed findings from the enemas and delayed radiographs. Examinations were performed under fluoroscopy with either diluted barium or water-soluble contrast media. Findings evaluated in every study were as follows: TZ, rectosigmoid index (RI), bowel wall irregularity, and irregular contractions, filling defect due to fecal material and delay in contrast evacuation after 24 hours. RI was obtained by dividing the widest diameter of the rectum by the widest diameter of the sigmoid loop when the colon was fully distended by the contrast medium. The normal RI is ≥ 1, and in HD, the RI is ≤ 1. A delayed film was labelled positive if the contrast was not completely evacuated and when the residual contrast was present up to the transverse colon, not beyond that.

Full-thickness biopsy was obtained by an experienced pediatric surgeon from one location 2 cm above the dentate line. A board-certified pathologist blindly reviewed the pathology reports of the samples from the colorectal biopsy along with the final specimens. A positive biopsy was defined as the absence of ganglion cells and the presence of nerve fiber hypertrophy. Biopsy samples, which were reported as to be equivocal or insufficient, were taken as negative. 

Data analysis was performed using Statistical Package for Social Sciences (SPSS v.19, IBM Corp. in Armonk, NY). Specificity and sensitivity along with positive predictive value (PPV) and negative predictive value (NPV) were calculated for each finding according to the rectal biopsy as the gold standard.

## Results

The patient ages ranged from the youngest being three days to the oldest being 10 years with mean and median age of 4.2 and 6.5 years, respectively. Seventeen (21%) patients were female, and 65 (79%) were male. Thirty-six (44%) patients were of age one year or younger. Failure of the passage of meconium, abdominal distension, and constipation were the common presenting symptoms with frequencies of 36 (44%), 30 (36.5%), and 20 (24%), respectively.

**Figure 1:**
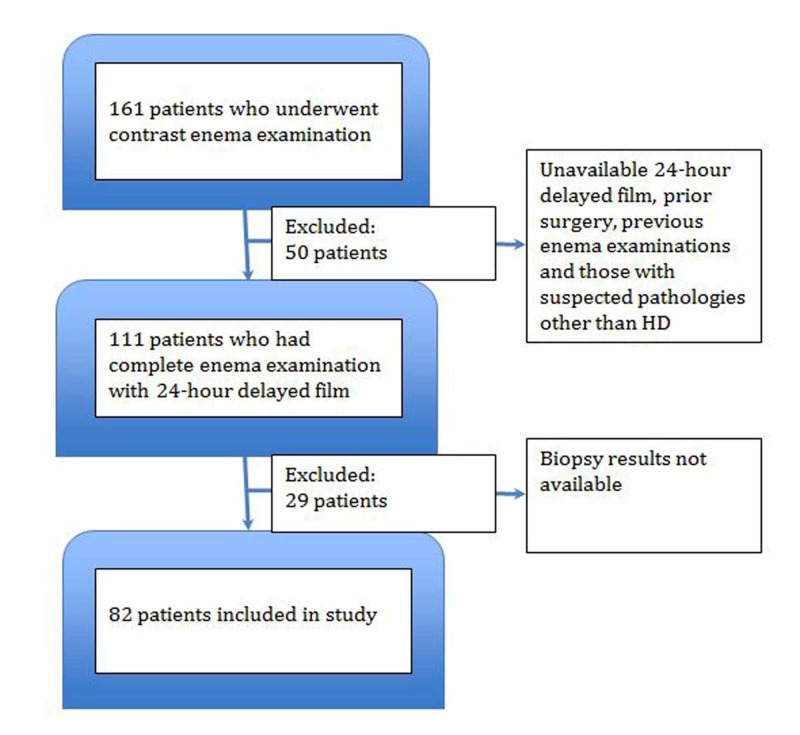
Flow chart depicting the inclusion of patients in the study HD, Hirschsprung’s disease

Out of the 160 patients who underwent CE examination during our study period, 111 met the inclusion criteria. Out of the 111 patients, only 82 were included in the study as they had both biopsy results and delayed 24-hour films (Figure 1).

Patients were assigned to the HD or non-HD group, depending on the pathology result of rectal biopsy. HD was confirmed using rectal biopsy in 56 (43 patients were male and 13 were female) of 82 cases (Table 1).

**Table 1:** Frequency of positive findings on contrast enema among patients with and without HD on biopsy HD, Hirschsprung's disease

	HD positive on biopsy (56)	HD negative on biopsy (26)
Positive on contrast enema	45	7
Negative on contrast enema	11	19

**Figure 2:**
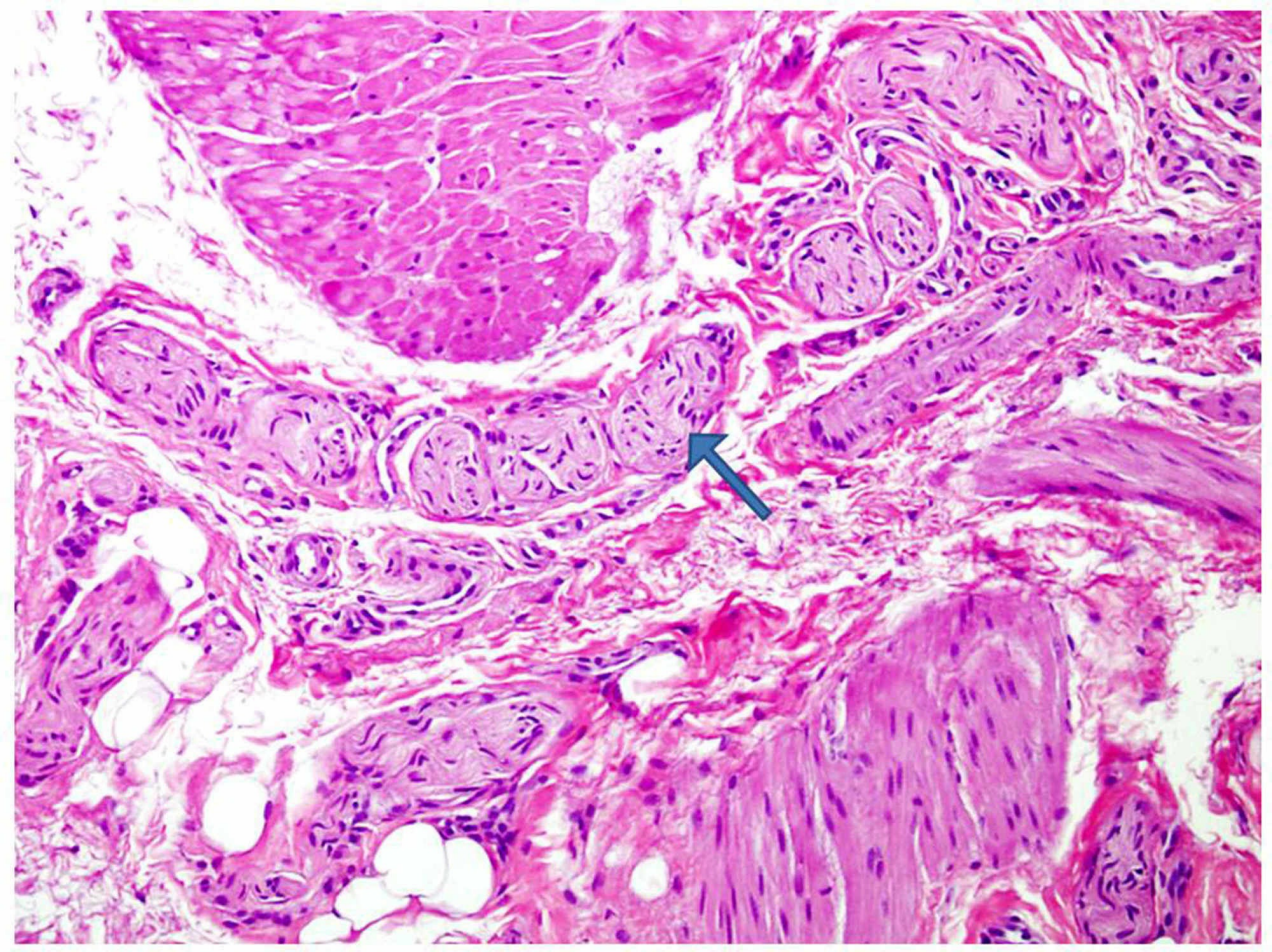
Numerous hypertrophic nerve bundles (arrow) were seen within muscularis propria fibers (H&E 200x magnification)

Histologically, in HD cases, numerous hypertrophic nerve bundles were seen within sub-mucosa and muscularis propria (Figure 2). In HD-negative cases, normal mature ganglion cells were noted, and in most of the cases, these were few in number.

The overall sensitivity, specificity and PPV of CE was 80.36% (confidence interval [CI]: 67.57% to 89.77%), 73.08% (CI: 52.21% to 88.43%), and 86.54% (CI: 77.11% to 92.46%), respectively.

**Figure 3:**
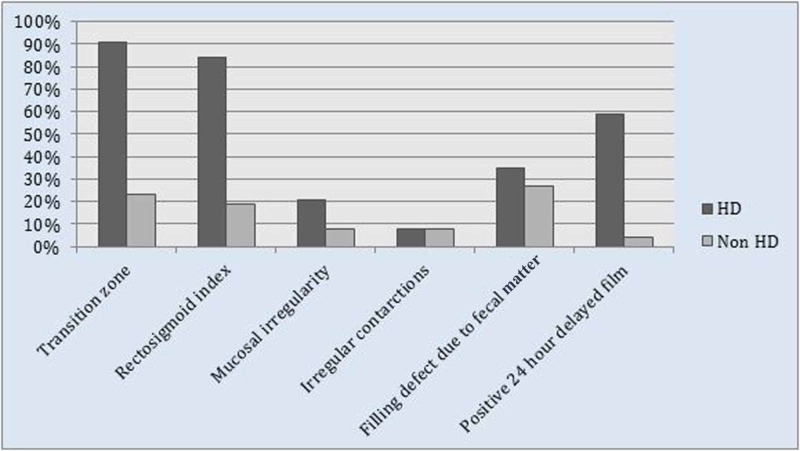
Frequency of radiological findings on contrast enema among patients with and without HD on biopsy HD, Hirschsprung’s disease

TZ was identified in 51 cases, being the most commonly seen CE examination finding in patients diagnosed with HD (Table 2). The most sensitive radiological finding was also the TZ with a sensitivity of 91.07% (Table 2). In all, 59% patients had positive delayed 24-hours film and were confirmed having HD on biopsy (Figure 3).

**Table 2:** Frequency of radiologic findings in CE among patients with and without HD on biopsy CE, contrast enema, HD, Hirschsprung's disease

		HD positive on biopsy (56)	HD negative on biopsy (26)
Transition zone (TZ)	Positive on CE	51 (91%)	6 (23%)
	Negative on CE	5 (9%)	20 (77%)
Rectosigmoid index (RI)	Positive on CE	47 (84%)	5 (19%)
	Negative on CE	9 (16%)	21 (81%)
Bowel wall irregularity	Positive on CE	12 (21%)	2 (8%)
	Negative on CE	44 (79%)	24 (92%)
Irregular contraction	Positive on CE	5 (8%)	2 (8%)
	Negative on CE	51 (92%)	24 (92%)
Filling defect due to fecal material	Positive on CE	20 (35%)	7 (27%)
	Negative on CE	36 (65%)	19 (73%)
Delay in contrast evacuation after 24 hours	Positive on CE	33 (59%)	1 (4%)
	Negative on CE	23 (41%)	25 (96 %)

**Figure 4:**
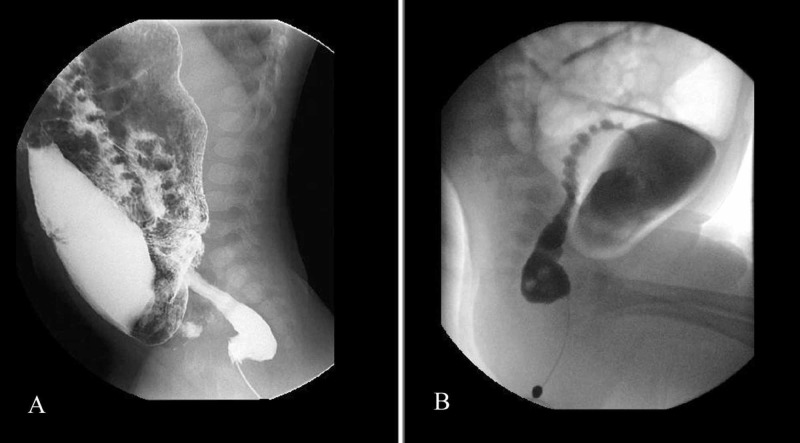
Spot images (A & B) of contrast enema showing an abnormal rectosigmoid index (<1), dilated sigmoid colon and narrow rectum.

RI was the second most common finding on CE with a sensitivity and specificity of 91.07% and 83.93%, respectively, as shown in Table 3 and Figure 4. Out of 33 patients who had positive 24-hour film and HD on biopsy, 20 patients were older than one year of age and 13 patients were one year or younger. In 76% of the patients younger than one year, the procedure was performed with non-ionic contrast, whereas in 56% percent of older patients, the procedure was performed with non-ionic contrast.

**Table 3:** Summary of sensitivity and specificity of radiological findings in HD CE, contrast enema; HD, Hirschsprung's disease; CI, confidence interval

Finding on CE	Sensitivity and specificity of radiological findings in HD	
	Sensitivity	Specificity
Transition zone (TZ)	91.07 % (CI: 80.38% to 97.04%)	76.92 % (CI: 56.35% to 91.03%)
Rectosigmoid index (RI)	83.93 % (CI: 71.67% to 92.38%)	80.77 % (60.65% to 93.45%)
Bowel wall irregularity	21.43 % (CI: 11.59% to 34.44%)	92.31 % (CI: 74.87% to 99.05%)
Irregular contraction	8.93% (CI: 2.96% to 19.62%)	92.31 % (CI: 74.87% to 99.05%)
Filling defect due to fecal material	35.71% (CI: 23.36% to 49.64%)	73.08 % (CI: 52.21% to 88.43%)

**Figure 5:**
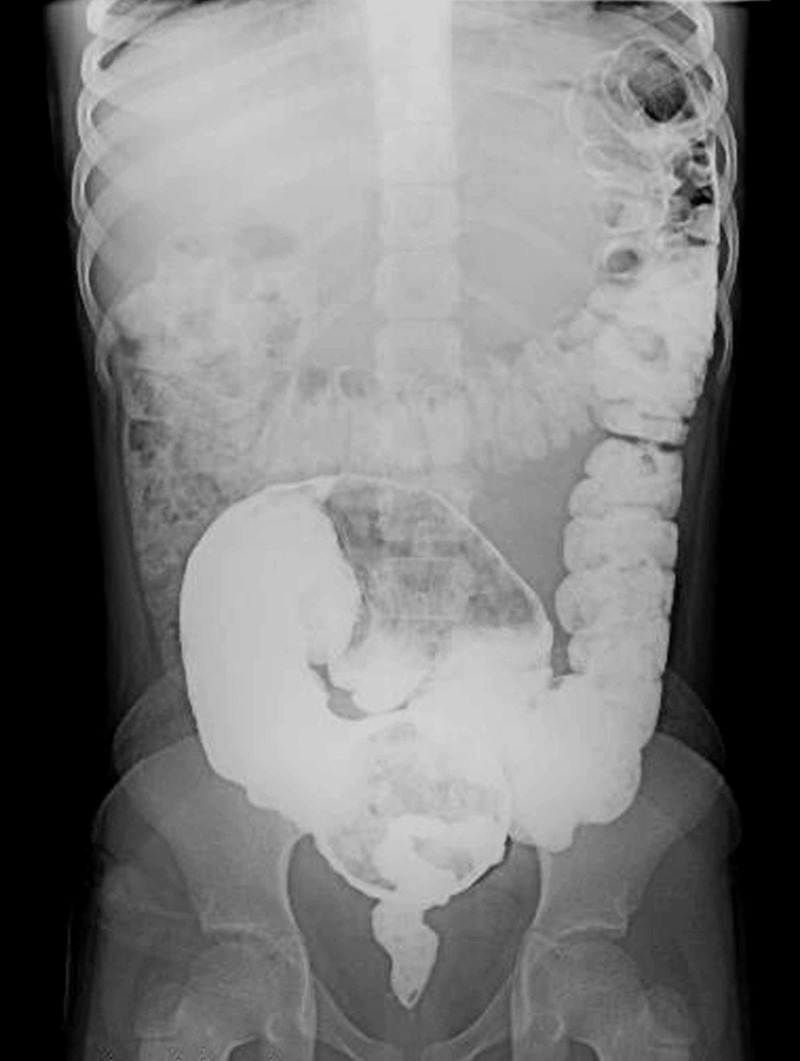
Retained contrast in the large bowel on the 24-hour delayed film after contrast enema examination

The sensitivity, specificity and PPV of delay in contrast evacuation after 24 hours with other positive findings on CE was 81.25% (CI: 67.37% to 91.05%), 90.91% (CI: 58.72% to 99.77%) and 97.50% (CI: 85.69% to 99.61%) respectively (Table 4). In 12 patients with high clinical suspicion of HD, 24-hour delayed film was the only positive finding (Figure 5), out of which HD was confirmed with rectal biopsy in eight patients.

**Table 4:** Sensitivity and specificity of delay in contrast evaluation after 24 hours in HD CE, contrast enema; HD, Hirschsprung's disease; CI, confidence interval

Finding on CE	Sensitivity and specificity of radiological findings in HD	
	Sensitivity	Specificity
Delay in contrast evacuation after 24 hours + other positive findings on CE	81.25% (CI: 67.37% to 91.05)	90.91 % (CI: 58.72% to 99.77%)
Delay in contrast evacuation after 24 hours only	88.89% (CI: 51.75% to 99.72%)	33.33 % (CI: 0.84% to 90.57%)

## Discussion

Mortality from HD has seen a reduction from 80% to 13%; this trend is likely due to and highlights the role of early diagnosis [7]. Biopsy remains the gold standard for diagnosis; however, the first investigation is usually a CE examination [8-10]. In this study, our main aim was to evaluate the usefulness of the 24-hour delayed film in our center and validate the use of additional radiation that is given to the child via the delayed film. This is the largest study conducted on HD in a tertiary-care center of Pakistan to the best of our knowledge.

Two out of these patients were diagnosed with total aganglionosis and one with skip segments. Our results showed that almost all the cases with a long segment, skip segment or with aganglionosis showed positive 24-hours delayed film. These results showed concordance with a past study [11]. In 34 of the patients who had positive delayed film, approximately 20 patients had a positive TZ and 14 had a positive RI. We found a higher sensitivity of delayed film alone of 88.89% (CI: 51.75% to 99.7%), whereas combined with other radiological findings, the sensitivity was 81.25% (CI: 67.37% to 91.05). However, we found a lower specificity of 33.33% (CI:0.84% to 90.57%) of the 24-hour delayed film alone, indicating that there may be other causes of retained contrast such as functional constipation.

In disparity to past studies done by Chun et al., our criteria of a positive 24-hour delayed film was a little different as we considered positive film when the residual contrast was present up to the transverse colon and not beyond that [6,11]. This also needs to be investigated in future studies, because developing one single criteria or definition for positive 24-hour delayed film could have an effect on diagnostic values of enema examination.

We found the sensitivity of the delayed film to be 81.25% which is similar to data from prior studies that have shown sensitivities between 60% to 99% [9-12]. With respect to specificity and PPV, in comparison to previous studies, we obtained a high value of 90.9% and 97.5%. In a previous study, specificity and PPV were reported as 7.6% and 20.6%, respectively, while another study described the specificity and PPV to be 73.7% and 83.9% [9,12].

The overall sensitivity and specificity of CE examination for detection of HD were 80.36% (CI: 67.57% to 89.77%) and 73.08% (CI: 52.21% to 88.43%), respectively. Reported sensitivity and specificity of enema examination in the diagnosis of HD have shown a considerable range of 60-100 % [13-15]. Enema examination has a sensitivity (65% to 80%) and specificity (66% to 100%) than rectal biopsy which has the highest sensitivity (91% to 100%) and specificity (97% to 100%) [11].

Studies have shown the TZ and RI as the most common signs on CE, as seen in our study as well (Figure 5) [16]. The most sensitive radiological finding in our study was the TZ, with the usual location being the rectosigmoid region followed by positive RI with sensitivities of 91.07% and 83.93%, respectively. This is likely because of the pathophysiology of the disease. These two features were also well correlated with histopathology and intraoperative findings in past studies [6,11]. 

There are, however, different factors that do not enable demonstration of the TZ, with the most common being the presence of colitis in which the spastic colon does not permit the formation of the TZ and a spiculated mucosa is observed. Furthermore, using a catheter with an inflated balloon, inserting a long segment of the catheter, injecting the contrast agent at high pressure, improper radiological technique, and rapid filling of the colon do not enable demonstration of the TZ. CE in HD is helpful in planning the surgical procedure as it locates the TZ. However, it must be kept in mind that it has been shown to be unable to determine the exact length of the affected segments [17-19]. Different studies have however shown the length of the TZ to be ≤5 cm [20-23]. Recent trends have shown that pediatric surgeons use contrast enema only in positive cases after rectal biopsy-proven HD with the goal to demonstrate the TZ [24]. In our country, however, most patients present after the neonatal period with episodes of functional constipation, therefore we make use of the CE as a screening tool to rule out HD.

There are a few limitations to our study. Because of the retrospective nature of this study, we have no knowledge about the exact amount of rectal contrast given to these children, as the amount of contrast could have an effect over the residual amount of contrast in 24-hour delayed film. Also, 76% of the patients, especially neonates, in our study were given water-soluble contrast instead of barium. We did not evaluate the difference in the diagnostic accuracy, if any, of the enema examination and the 24-hour delayed film depending on the administration of either barium or non-ionic contrast.

## Conclusions

Contrast enema examinations along with the 24-hour delayed film with mid-transverse colon cut-off are optimal for initially investigating HD, especially in a developing nation where it is usually used as a first-line investigation. Our results show that it correlates strongly with biopsy as well. However, rectal biopsy still remains the gold standard for diagnosis. Finally, the level of TZ once demonstrated is also a useful predictor for the actual disease involvement and for planning surgical intervention.
